# Kocher-Debre-Semelaigne Syndrome: Hypothyroid Muscular Pseudohypertrophy—A Rare Report of Two Cases

**DOI:** 10.1155/2012/153143

**Published:** 2012-03-12

**Authors:** Chandan Shaw, Prachi Shaw

**Affiliations:** ^1^Department of Pediatrics, Sri Manakula Vinayagar Medical College and Hospital, Kalitheerthalkuppam, Madagadipet, Pondicherry 605 107, India; ^2^Department of Microbiology, Sri Manakula Vinayagar Medical College and Hospital, Kalitheerthalkuppam, Madagadipet, Pondicherry 605 107, India

## Abstract

Kocher-Debre-Semelaigne syndrome (KDSS) is a rare association of muscular pseudohypertrophy and long-standing moderate-to-severe hypothyroidism in the pediatric age group. It may be confused with primary muscle disorders, lest one is cautious enough to investigate for hypothyroidism. The striking clinical features, availability of a simple treatment and a good prognosis for the condition makes it worthwhile to report the case so that all practitioners be aware of the condition and its management.

## 1. Introduction

 Kocher-Debre-Semelaigne syndrome (KDSS) is a rare association of muscular pseudohypertrophy and long-standing moderate-to-severe hypothyroidism in the pediatric age group. The condition is rare in countries with screening programmes for hypothyroidism at birth. However, they are not uncommon in the countries where such routine screening programmes are not available, the diagnosis of hypothyroidism may be delayed—which may account for the higher incidence of the said condition.

## 2. Case Report

**Figure 1:**
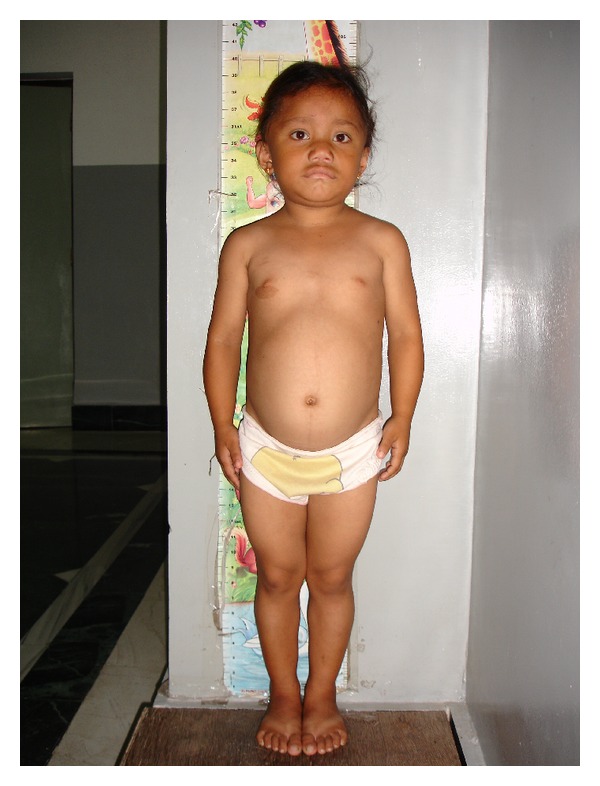
A 9-year-old girl with pseudomuscular hypertrophy in Kocher-Debre-Semelaigne syndrome.

**Figure 2:**
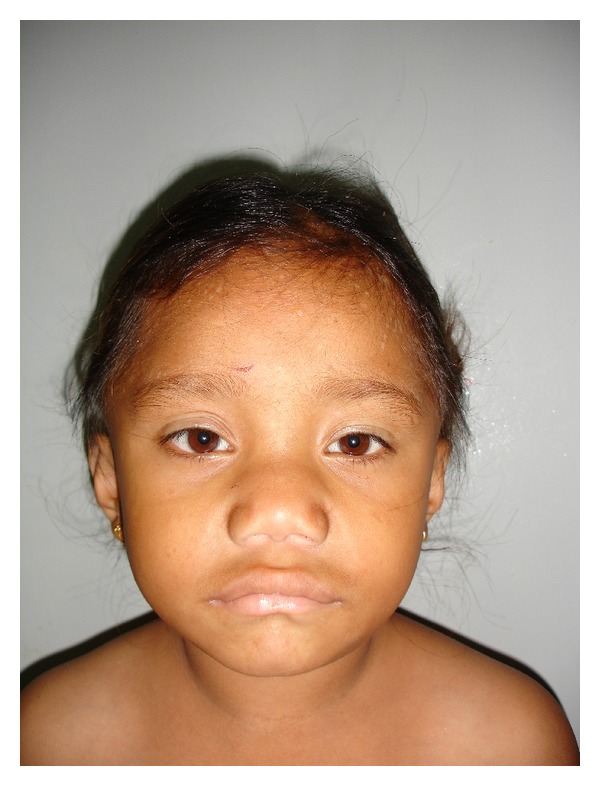
Facial features in the same girl with Kocher-Debre-Semelaigne syndrome.

 A 9-year-old girl, hailing from Senji, a village in Pondicherry, India, presented with lassitude, lethargy, mental slowing, and growth failure to the Department of Pediatrics, Sri Manakula Vinayaga Medical College and Hospital, Pondicherry. The child was apparently asymptomatic till 5 years of age, when the parents started noticing progressive weakness and lethargy along with deteriorating academic performance. She had difficulty in memorising lessons and performing mathematical calculations. There was no history of any neck swelling at any point in the history. There was no major illness reported prior to the onset of these symptoms. The milestones were achieved in the normal periods and no regression was noted. There was no similar illness, consanguinity or a history of neck swelling in the family and the dietary history was insignificant. On examination she was found to be grossly short statured [height  =  100 cms against expected of 132.5 cms (75.5%)] with prominent muscular build but with infantile proportions (Figure 1). She had hoarseness of voice, a dry texture of hair and skin, and infantile facies with macroglossia and pouting lips (Figure 2). Her systemic examination revealed low intelligence quotient with psychomotor retardation, decreased power in all four limbs involving both proximal and distal muscles (grade 3/5 to 4/5), hung-up knee jerk, and a ejection systolic murmur at the base of the heart. On investigation, she was found to have hypothyroidism [TSH = 115.6 IU/L (0.2–6.0 IU/L), total T_4_ = 0.12 *μ*g/dL (5–12.5 *μ*g/dL), T_3_ = 4.4 ng/dL (60–180 ng/dL)]. The creatinine phosphokinase (CPK), serum aspartate transaminase (AST), and the lactate dehydrogenase (LDH) levels were elevated. Muscle biopsy (light microscopy/H and E staining) revealed degenerative changes with hyalinization, vacuolization, and fragmentation of fibres without any inflammatory infiltrate suggestive of myopathy. Echocardiography revealed no cardiac abnormality. A diagnosis of KDSS was made on the basis of the above mentioned findings, and the child was started on levothyroxine supplementation, 50 *μ*g/day, three months following which the child was found to be euthyroid [TSH = 2.7 IU/L, T_3_ = 75 ng/dL, and T_4_ = 8.6 *μ*g/dL], and the symptoms of hypothyroidism had regressed but the muscular hypertrophy still persists after one year of thyroxine supplementation.

**Figure 3:**
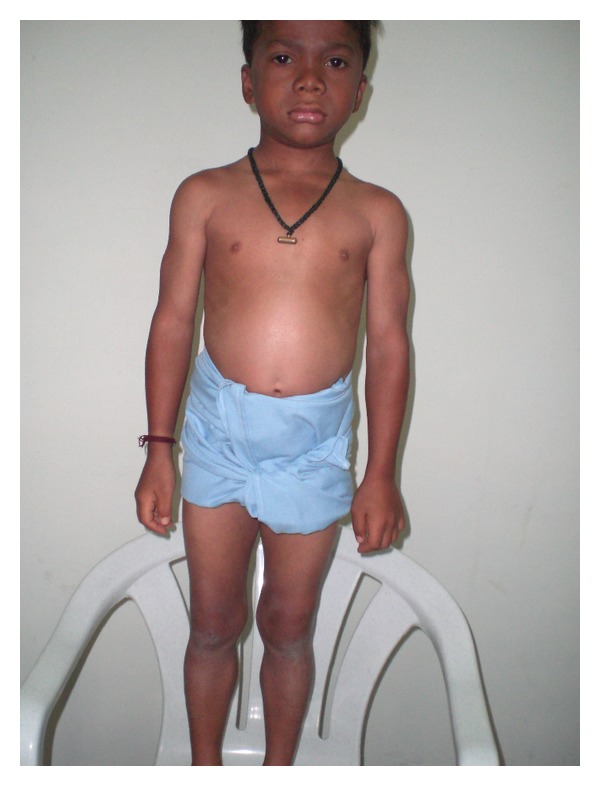
A ten-year-old boy with pseudomuscular hypertrophy in Kocher-Debre-Semelaigne syndrome.

**Figure 4:**
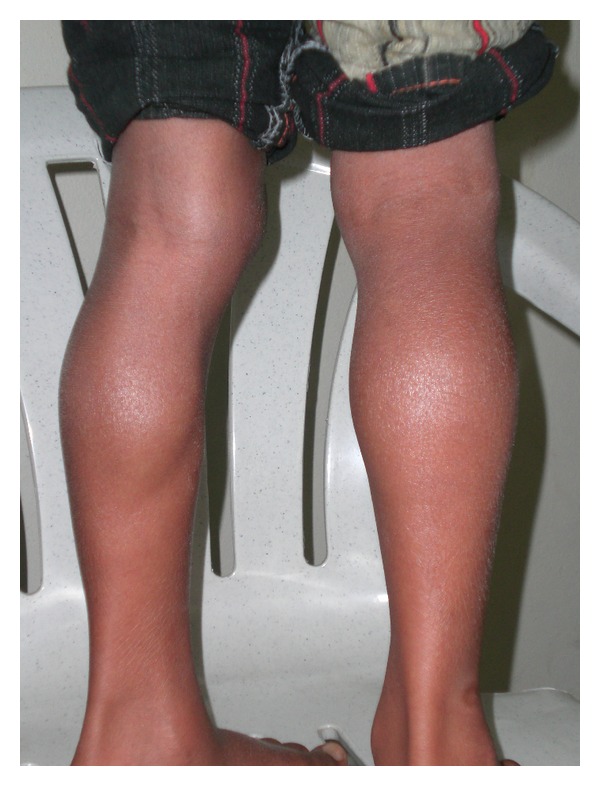
Calf pseudohypertrophy in the same boy with Kocher-Debre-Semelaigne syndrome.

 Another case is a ten-year-old boy hailing from Narketpally (Andhra Pradesh, India) with similar symptoms and signs (Figures 3 and 4) presented to our unit. The child had been symptomatic with symptoms like lethargy, cold intolerance, and dry skin coarse facial features for the last 6 years. The thyroid function tests were suggestive of hypothyroidism [TSH = 95.6 IU/L (0.2–6.0 IU/L), total T_4_ = 0.16 *μ*g/dL (5–12.5 *μ*g/dL), and T_3_ = 19.4 ng/dL (60–180 ng/dL)]. The CPK, AST, and muscle biopsy showed similar changes as the first child. The child was started on levothyroxine (50 *μ*g/day), and within one month the symptoms resolved though the muscular hypertrophy still persists after nine months of thyroxine supplementation. The child was euthyroid after 3 months [TSH = 4.7 IU/L, T_3_ = 65 ng/dL, T_4_ = 6.6 *μ*g/dL] Both the children are under followup.

## 3. Discussion

 The syndrome was initially reported by Emil Theodore Kocher in 1892, while the coexistence of the muscular pseudohypertrophy was emphasized by Robert Debre and Georges Semelaigne only later in 1935 [1]. The pathogenesis of muscular pseudohypertrophy remains obscure and is believed to be due to the long-standing effects of hypo-thyroidism on the muscle fibres. The histology is nonspecific. The light microscopic features are similar to that seen in the index case; additionally one may find variation of fibre size and abortive spiral annulets [2, 3]. KDSS is usually seen in the age group 3 to 10 years [1–4]; however, there have been anecdotal reports of cases presenting as early as in the neonatal period [5] and 15 months [4]. The underlying thyroid effect may vary from both congenital (athyreosis, enzyme synthesis defects) or acquired (autoimmune) forms of hypothyroidism [1, 6]. In either of the cases reported by us, the exact cause of hypothyroidism was not ascertained, though the ultrasonography of neck and aspiration cytology of the thyroid gland was normal, thus, ruling out athyreosis. The child usually has florid symptoms of hypothyroidism and severity of the latter usually correlates directly with the muscle pseudohypertrophy and muscle cramps. This is in stark contrast to the thyroid myopathy seen in hyper- or hypothyroid state in the adults wherein wasting and atrophy are more common. The smaller body proportions in children, lower fat content and myxedema impart an athletic or Herculean semblance [1–3]. The pseudohypertrophy is most striking in the limbs, tongue, and the facial muscles as seen in the reported case [1, 4]. One may be misled by a diagnosis of a primary muscle disorder but for the biochemical evidence of hypothyroidism. The elevation of CPK levels [7] are also to mild degree and not in the proportion seen in muscular dystrophies, which are the closest clinical differentials to the syndrome considering the age group. Besides, the dystrophic changes are not seen in biopsy. The electromyography shows a myogenic lesion [8]. An association of KDSS with congenital nystagmus has been reported in the literature, which was not seen in our case. The signs and symptoms of hypothyroidism as well as the muscular pseudohypertrophy revert [3, 7] back to normal in due course of time after initiation of thyroxine supplementation, except for the fact that the final height may still be short. A well-planned and graded physiotherapy programme may be beneficial in getting rid of the muscle stiffness and achieving full potential for the muscle strength [7].

## 4. Conclusion

 KDSS is a rare case which may be confused with primary muscle disorders, lest one is cautious enough to investigate for hypothyroidism. The striking clinical features, availability of a simple treatment, and a good prognosis for the condition makes it worthwhile to report the case so that all practitioners are aware of the condition and its management.
